# Supplementary material for: The interactome of intact mitochondria by cross-linking mass spectrometry provides evidence for coexisting respiratory supercomplexes
Source: Mol Cell Proteomics. 2017 Dec 8;17(2):216–32. doi: 10.1074/mcp.RA117.000470 (PMC5795388; doi:10.1074/mcp.RA117.000470)
Supplement: Supplemental Data [file supp_RA117.000470_133922_0_supp_23978_fzffwf.zip › spectra_annotation/mito_spectra_annotation/342-1-7-1-8-1.pdf]

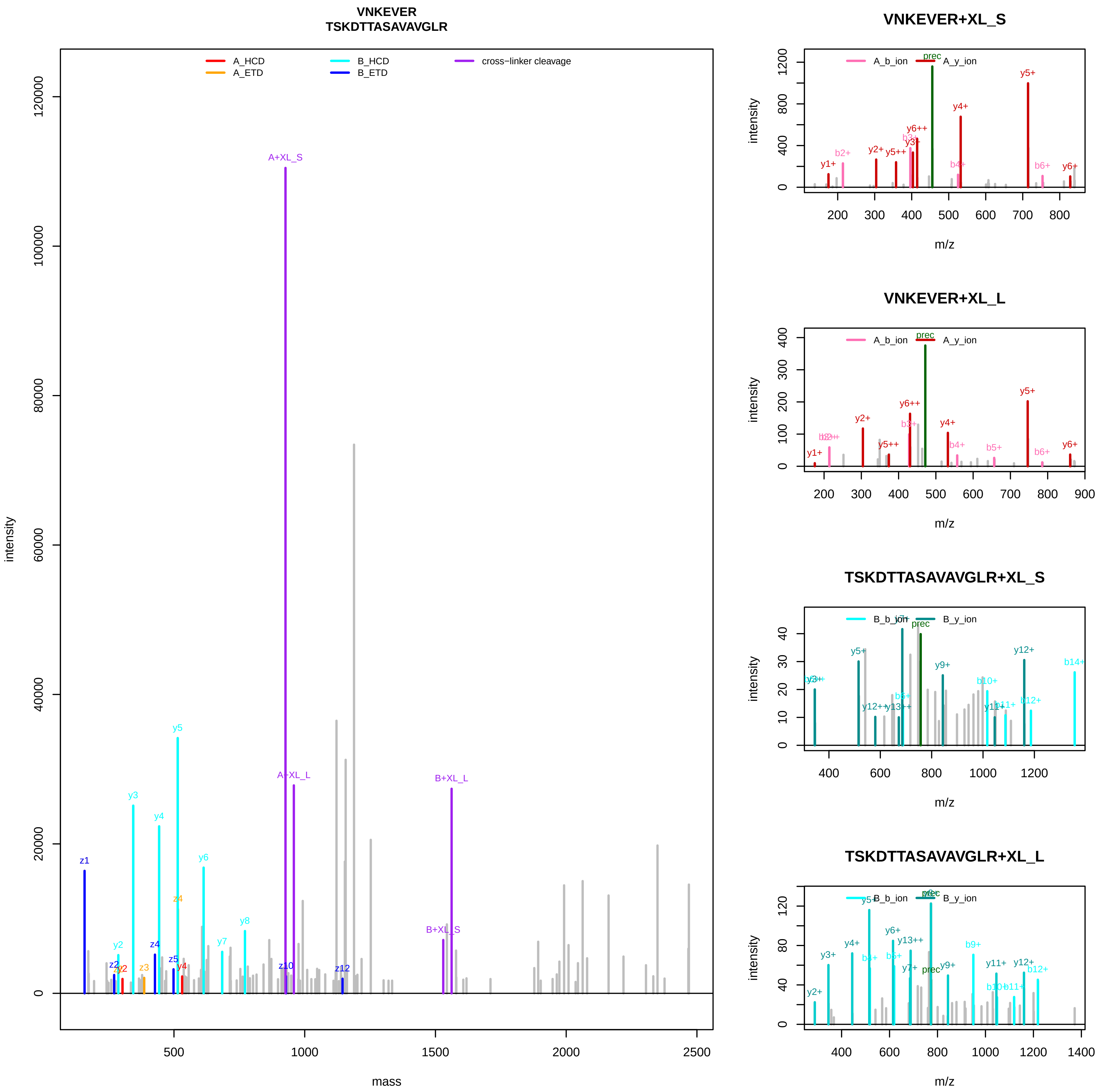
VNKEVER
TSKDTTASAVAVGLR
A_HCD
A_ETD
B_HCD
B_ETD
cross−linker cleavage
A+XL_S
A+XL_L
B+XL_L
B+XL_S
z1
z2
y2
z3
z4
y3
y4
y5
y6
y7
y8
z5
z10
z12
intensity
mass
0
20000
40000
60000
80000
100000
120000
500
1000
1500
2000
2500
VNKEVER+XL_S
A_b_ion
A_y_ion
prec
y1+
b2+
y2+
y5++
y6++
y3+
y4+
y5+
b4+
b6+
y6+
intensity
m/z
VNKEVER+XL_L
TSKDTTASAVAVGLR+XL_S
B_b_ion
B_y_ion
y5+
y9+
y12+
b10+
b12+
b14+
TSKDTTASAVAVGLR+XL_L
y2+
y3+
y4+
y6+
y13++
b9+
y11+
b12+
